# Comparison of contrast-dependent phase sensitivity in primary visual cortex of mouse, cat and macaque

**DOI:** 10.1097/WNR.0000000000001307

**Published:** 2019-08-30

**Authors:** Molis Yunzab, Shaun L. Cloherty, Michael R. Ibbotson

**Affiliations:** a National Vision Research Institute, Australian College of Optometry, Carlton; b Department of Optometry and Vision Sciences, University of Melbourne, Parkville; c Department of Physiology, Monash University, Clayton, VIC, Australia

**Keywords:** complex cell, mouse V1, phase sensitivity, primary visual cortex, visual system

## Abstract

Neurones in the primary visual cortex (V1) are classified into simple and complex types. Simple cells are phase-sensitive, that is, they modulate their responses according to the position and brightness polarity of edges in their receptive fields. Complex cells are phase invariant, that is, they respond to edges in their receptive fields regardless of location or brightness polarity. Simple and complex cells are quantified by the degree of sensitivity to the spatial phases of drifting sinusoidal gratings. Some V1 complex cells become more phase-sensitive at low contrasts. Here we use a standardized analysis method for data derived from grating stimuli developed for macaques to reanalyse data previously collected from cats, and also collect and analyse the responses of 73 mouse V1 neurons. The analysis provides the first consistent comparative study of contrast-dependent phase sensitivity in V1 of mouse, cat and macaque monkey.

## Introduction

The receptive fields (RFs) of V1 cells are classified as simple or complex based on the spatial structures of their RFs[1,2] and their responses to drifting sinusoidal gratings [3–5]. Simple cells have segregated subfields that detect either brightness increments (ON) or decrements (OFF) [1]. Complex cells are far less phase-sensitive than simple cells [1,2,6–10]. When stimulated with drifting gratings, simple cell spiking responses oscillate at the fundamental frequency of the stimulus, that is, spikes occur during one phase of the cycle (ON or OFF) and few spikes are present in the opposite phase. Spiking responses of complex cells are generated during both the ON and OFF phases [10].

Studies in cats and monkeys using drifting gratings showed that some V1 neurons have phase invariant (complex) responses at high stimulus contrasts but phase-sensitive responses at low contrasts (cat [11,12], monkey [13,14]). An intracellular study on 21 mouse V1 cells (simple: 14; complex: seven) revealed similar flexibility in the phase sensitivities of subthreshold membrane potential responses [15]. These studies used different techniques to investigate the phase sensitivity of V1 neurons. Here, to increase the cell population and to directly compare mouse data with previous studies, we collected data from 73 mouse V1 neurons, of which 63 were complex cells, using extracellular recording. Using the analysis introduced for macaques [14], we also reanalysed 416 neurons from areas 17 and 18 in cats. These areas each contain complete visual field representations, and both receive direct input from the thalamus, which defines an area as primary visual cortex. Therefore it appeared appropriate to compare these areas collectively against the cells from the primary visual cortices of monkeys and mice [16]. As a result, we provide the first comparison with a single analysis technique of contrast-dependent phase sensitivity in mouse, cat and primate.

## Methods

### Electrophysiology

Extracellular recordings were made from C57BL/6 mice (5–12 weeks old). Experiments were approved by Melbourne University’s Animal Ethics Committees (Yunzab *et al.*, 2019). Mice were anesthetised with intraperitoneal injections of chloroprothixene (10 mg/kg) followed by urethane (1 g/kg). The level of anaesthesia was monitored using the electrocardiogram and toe-pinches. Body temperature was kept at 37°C using an auto-regulating heat blanket. A tracheotomy was performed to ensure a clear airway and a craniotomy (1 × 2.5 mm) opened over V1. Recordings were made with gold-tipped, lacquer-coated tungsten electrodes (impedance 1–2 MOhms; FHC, Bowdoinham, Maine, USA). Signals were amplified, band-pass filtered (300 Hz–6 kHz) and sampled at 40 kHz using a CED 1401 interface and Spike2 software (Cambridge Electronic Designs, Cambridge, UK). Spikes were identified using a Schmitt trigger. Units were recorded 150–700 μm beneath the cortical surface.

### Stimulus protocol

Visual stimuli were generated with a ViSaGe stimulus generator (Cambridge Research Systems, Cambridge, UK) and displayed on a calibrated CRT monitor (Clinton monoray, 100 Hz non-interlaced refresh rate, 1024 × 768 pixels, 57 cd/m^2^ mean luminance). Viewing distances were 30 cm for mice and 57 cm for cats [17] and 114 cm for macaques [14]. For each recorded cell, the preferred temporal and spatial frequency (TF, SF), orientation, location and size of the RF were determined with drifting gratings at 100% contrast. Michelson contrast = [(Lum_max_ − Lum_min_)/Lum_max_ + Lum_min_)] × 100, where Lum_max_ and Lum_min_ are the maximum and minimum grating luminance.

Stimuli were drifting sinusoidal gratings with optimal TF, SF and orientation presented in a circular aperture the size of the RF. Receptive fields were within 2–5° eccentricity in monkeys and up to 7° eccentricity in cats and mice. Drifting gratings with contrast of 0%–100% were presented in pseudorandom order interleaved with 1 second blank periods (mean luminance). Gratings were presented for 3 seconds: first and last 0.5 seconds stationary; drifting in between.

### Response analysis

The mean firing rate for each stimulus condition was calculated by cycle-averaging the response across trials. Spontaneous activity was calculated by averaging the firing rate in the 500 ms period before each stimulus presentation. Phase sensitivity was quantified using the F_1_/F_0_ ratio, where F_1_ is the amplitude of the Fourier coefficient at the grating’s fundamental frequency and F_0_ is the mean firing rate above spontaneous. Fourier coefficients were calculated using the FFT function in Matlab (The Mathworks Inc., USA). For each cell, the F_1_/F_0_ ratios at high and low stimulus contrasts were compared. The high contrast condition generated the highest firing rate. Because of differences in contrast gain, low contrast conditions varied between cells. A Poisson distribution was calculated from the spontaneous firing rate and a response threshold determined by the 99% confidence limit of the Poisson distribution [11]. The lowest stimulus contrast that generated a response above the Poisson threshold was the low contrast condition.

As the F_1_/F_0_ ratio is sensitive to the number of spikes [10,11], we compared the observed ratio with an empirical distribution of F_1_/F_0_ from a simulated complex cell using the analysis described in Cloherty and Ibboston [14]. We assigned the model complex cell to produce *n* spikes over the full sinusoidal cycle. The spike arrival times, *t*_*i*_ ε (−*π*,*π*),*i* = 1...*n*, were assumed to be independent and identically distributed values randomly drawn from a raised cosine defined by,


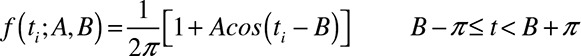
(1)

where A (0 ≤ *A* ≤ 1) represents the assumed true or asymptotic value of F_1_/F_0_ as *n* → ∞ and B defines the position of the distribution. We simulated spike arrival times (*t*_*i*_) using Equation 1 for a chosen asymptotic F_1_/F_0_ and the position where *A* = (*F*_1_/*F*_0_)_∞_ and *B* = 0. The asymptotic F_1_/F_0_ was estimated for each cell by maximizing the likelihood of the observed data. The log-likelihood (L) of the data for a given asymptotic F_1_/F_0_ was computed using:


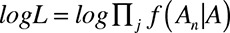
(2)

where *A*_*n*_ is the F_1_/F_0_ value based on the cell’s actual spike count (*n*), A is the assumed asymptotic F_1_/F_0_ for the simulation and j indicates the contrast at the maximal response.

Using the asymptotic F_1_/F_0_ that maximized the likelihood of the high contrast data, we simulated responses with spike count (*n*) observed at the low contrast condition (Equation 1) and computed an empirical distribution of F_1_/F_0_. The increase in observed F_1_/F_0_ at the low contrast was significant only if it exceeded the 99% confidence limit of the empirical distribution [14].

## Results

### Mouse V1

**Fig. 1:**
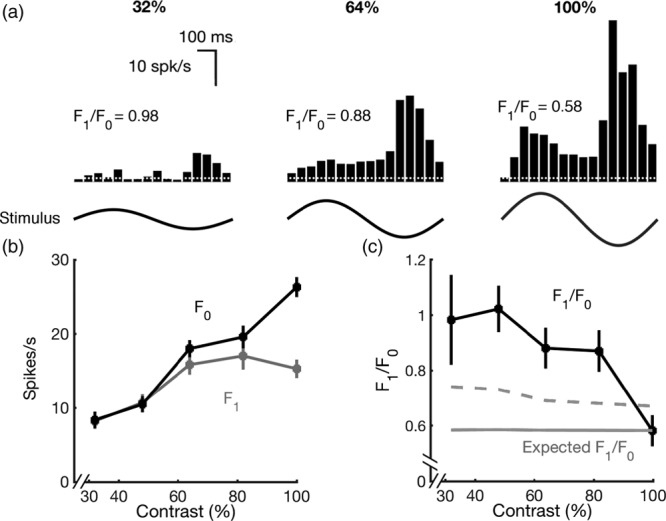
(a) Cycle-averaged spiking rates at contrast levels of 32%–100% for a V1 cell. The dashed lines show the spontaneous spike rates. Bottom panel: a visual representation of one cycle of the sinusoidal grating stimulus. (b) Amplitudes (spikes/s) of the modulated component of the responses (F_1_, grey line) and the mean responses (F_0_, black line) as functions of stimulus contrast for the same cell. (c) F_1_/F_0_ ratios as a function of stimulus contrast (black line). The light grey line shows the expected F_1_/F_0_ derived from the simulated empirical distributions for spike counts (red dashed line, 99% confidence limit of the empirical distributions). In (b and c), symbols indicate means and error bars show bootstrap estimates of standard error.

Recordings were obtained from 73 V1 neurons in 22 mice. Mean responses over a full cycle for an example complex cell at three contrast levels are shown (Fig. 1a). As contrast is reduced, the F_1_/F_0_ ratios increase. The amplitudes of the F_0_ and F_1_ components are also plotted as functions of stimulus contrast (Fig. 1b). Compared to F_1_, F_0_ decreases at a higher rate as contrast reduces, resulting in an increased F_1_/F_0_ (black line, Fig. 1c). As the number of spikes is less at lower contrasts, the F_1_/F_0_ is expected to increase even without a physiological mechanism. Figure 1c shows the F_1_/F_0_ ratio (red line) that is expected even without a physiological mechanism. It also presents the threshold (99% confidence limit) above which any change in F_1_/F_0_ is regarded as being caused by an additional physiological change (red dashed line). The observed F_1_/F_0_ ratio of the cell exceeds the threshold level for all contrasts except 100%. Therefore, we consider these changes in F_1_/F_0_ to not be due to a simple reduction in spike count.

**Fig. 2:**
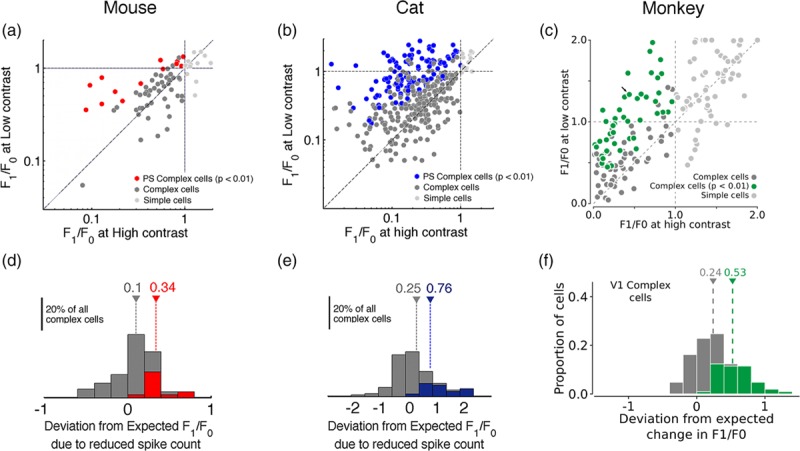
Population results of F_1_/F_0_ for V1 cells recorded with drifting gratings in mouse, cat and macaque at low compared to high stimulus contrasts. (a) Scatter plot of F_1_/F_0_ ratios for 73 mouse cells, (b) 416 cat cells and (c) 166 macaque cells (figure taken from Figure 4 of Cloherty and Ibbotson [14]). Complex cells that exhibit a significant increase (*P* < 0.001) in F_1_/F_0_ at low contrasts are in red (mouse), blue (cat) and green (macaque). Medium grey symbols represent the remaining complex cells. Simple cells shown with light grey symbols. (d–f) Distributions of changes in observed F_1_/F_0_ between low and high contrasts after subtracting the expected changes in F_1_/F_0_ due to low spike numbers. The coloured bars represent the distribution of the subset of complex cells that showed significant changes in F_1_/F_0_ at low contrast.

Figure 2a plots the F_1_/F_0_ observed at low contrast against the F_1_/F_0_ observed at high contrast for all V1 cell. Cells were classified as simple or complex based on their F_1_/F_0_ ratios at high contrast (simple = F_1_/F_0_ > 1, complex = F_1_/F_0_ < 1). Simple cells (light grey symbols, n = 10) show no consistent changes in F_1_/F_0_ between low and high contrast conditions (difference = 0.23, two-sided Wilcoxon signed-rank test, *P* = 0.02). For complex cells, 13/63 units (21%) showed a significant increase in F_1_/F_0_ at low contrast (difference = 0.12, two-sided Wilcoxon signed-rank test, *P* = 0.003). The observed increase exceeds the 99% confidence limit of the expected increase (red symbols; Fig. 2a).

We quantified the change in the observed F_1_/F_0_ at high and low stimulus contrasts after subtracting the change in F_1_/F_0_ expected due to the reduction in spike count. If the F_1_/F_0_ ratio observed at low contrast was due to spike count reduction, the distribution of the expected change is expected to be around 0. For all complex cells, this metric is significantly different from zero (difference = 0.1, two-sided Wilcoxon signed-rank test, *P* = 0.026; grey bars, Fig. 2d). The red bars in Fig. 2d show the distribution of the complex cells that exhibited significant increases in F_1_/F_0_ at low contrast. The mean of the distribution is significantly greater than zero (difference = 0.34, two-sided Wilcoxon signed-rank test, *P* < 0.001).

### Cat V1

We re-analysed a set of data collected from cat V1 that was published previously [11,12]. In cat, 114/365 complex cells (31%) showed an increase in F_1_/F_0_ ratio at low contrasts (Fig. 2b). The complex cells in cat V1 showed a significant increase in F_1_/F_0_ ratio (difference = 0.34, two-sided Wilcoxon signed-rank test, *P* < 0.001). The difference between the observed and expected increases in F_1_/F_0_ ratios lie significantly away from zero in cat V1 (difference = 0.25, two-sided Wilcoxon signed-rank test, *P* < 0.001, grey bars; Fig. 2e). Significant changes between the observed and the expected increases in F_1_/F_0_ ratio were also observed within the subset of cat V1 complex cells that showed increased F_1_/F_0_ ratios at low contrasts (difference = 0.76, two-sided Wilcoxon signed-rank test, *P* < 0.001, blue bars; Fig. 2e). The simple cells in cat V1 showed no significant changes in F_1_/F_0_ between low and high contrasts (difference = 0.08, two-sided Wilcoxon signed-rank test, *P* = 0.14; light grey symbols; Fig. 2b). Due to a deliberate selection bias towards complex cells in the original projects for which the data were collected, the cat data has a higher percentage of complex cells than appears in the monkey and mouse data.

### Macaque V1

The data in Fig. 2c and f exactly reproduces the data from Fig. 4a and e in Cloherty and Ibbotson [14], which introduced the analysis used here for mice and cats. In macaque V1 44% (46/105) of complex cells showed a significant increase in F_1_/F_0_ at low contrasts, whereas simple cells showed no consistent changes [14].

## Discussion

Contrast-dependent phase sensitivity has been identified in V1 of mouse, cat and macaque using different analysis, making direct comparison difficult [11,14,15]. A recent study by Yunzab *et al*. [15] conducted whole-cell intracellular recording from 21 mouse V1 cells using drifting gratings. Here we used single-electrode extracellular recording to obtain data from a further 63 mouse complex cells and revealed that 21% showed contrast-dependent phase sensitivity using the same analysis developed for macaque V1 [14]. We also re-analysed data collected from cat cortex, some of which have been published previously [11], using the same analysis. The cat data revealed that 31% of complex cells showed a significant increase in F_1_/F_0_ ratio at low contrasts. Although all three species showed that a proportion of cells revealed a clear increase in phase sensitivity at just-detectable contrasts the proportions of cells differed between species: macaques 44%, cats 31% and mouse 21%. It is noteworthy that drifting grating stimuli were used to assess the phase sensitivity in all three species. It has been suggested that the simple-complex dichotomy based on response modulation is stimulus-dependent [18,19]. One of the drawbacks of drifting gratings is their inability to separate the spatial and temporal components of response modulation. However, studies that employed contrast-reversing gratings, which allow measurements of spatial and temporal response components separately, also showed contrast-dependent phase sensitivity in all three species [15,20].

The level of contrast-dependent phase sensitivity may be influenced by mechanistic differences in the respective processing that occurs in the visual pathways between species. Cats and monkeys have a columnar organization of orientation selectivity in V1 [21,22], whereas mouse V1 neurons with different orientation preferences are intermingled randomly [23]. Orientation tuning is more common in mouse dorsal lateral geniculate nucleus (dLGN) neurons compared to neurons in cat and monkey dLGNs, which suggests differences in thalamic contributions to visual processing [24]. The emergence of direction selectivity along the visual pathways is also different between species. In the retina, the existence of direction-selective neurons is still uncertain in monkeys, whereas 20% of mouse retinal ganglion cells are direction-selective [25]. On the cortical level, direction-selective maps are present in area 18 in cat but absent in macaque V1 [26]. The differences in the hierarchies of orientation and direction selectivity processing through the respective visual pathways may influence how complex cells in cortex generate their phase invariance.

Governed by the ethical requirements imposed on each project, different anaesthetics were used. The macaque data were collected using opioid-based intravenous drugs (sufentanil [14]), the cat a mixture of gaseous Halothane (5%) and nitrous oxide (50% in pure oxygen [11]) and the mouse intraperitoneal injections of chloroprothixene (10 mg/kg) followed by urethane (1 g/kg). Urethane at the concentrations used by us has multiple effects on neurotransmitters [27]. Its main effects are to potentiate the functions of nicotinic acetylcholine, gamma-aminobutyric acid (GABA_A_), and glycine receptors, and inhibit N-methyl-D-aspartate and alpha-amino-3-hydroxy-5-methyl-4-isoxazole propionic acid receptors. The potentiation of GABA likely has a general inhibitory effect. The combination of chloroprothixene and urethane is widely used as the anaesthetic for *in vivo* mouse electrophysiology [15,28–30]. Halothane has multiple effects throughout the central nervous system. Particularly, it preferentially potentiates GABA_A_ receptors [31]. Nitrous oxide was also used in cats, which inhibits *N*-methyl-D-aspartate channels [31]. Opioids, such as sufentanil, generate increases in GABA release, amongst other effects [32]. In general, the enhancement of GABA is similar between anaesthetics but they may differ enough in detail to influence the percentage of contrast-dependent phase-sensitive cells in V1.

## Conclusion

All species showed contrast-dependent phase sensitivity in V1. Given the ease of using mice, it is good news that studies on this topic are viable in mice. However, the specie-differences in the proportion of cells showing the effect could result from different anaesthetic regimes. In addition, it must be acknowledged that differences in the neural networks and processing hierarchies in the different species may also have an affect.

## Acknowledgements

This work was supported by the Australian Research Council Centre of Excellence for Integrative Brain Function (CE140100007), the National Health and Medical Research Council (GNT0525459), the L.E.W Carty Charitable Fund & Lions Foundation of Victoria.

## Conflicts of interest

There are no conflicts of interest.
